# Improved outcomes of pediatric patients with swallowing disorders through a multidisciplinary dysphagia clinic in a tertiary care children's hospital in Colombia

**DOI:** 10.1002/pdi3.99

**Published:** 2024-07-10

**Authors:** Silvia J. Galvis‐Blanco, Víctor A. Martínez‐Moreno, Olga L. Morales‐Múnera, Alejandra Wilches‐Luna, Claudia L. Losada‐Gómez, Silvia Palacio‐Petri, Ángela M. Castañeda‐Agudelo, Janeth Rosero‐Vélez, Leidy J. Torres‐Pérez, Laura F. Niño‐Serna

**Affiliations:** ^1^ University of Antioquia Medellín Colombia; ^2^ Research Group on Diseases of Children and Adolescents (Pediaciancias), University of Antioquia Medellín Colombia; ^3^ San Vicente Fundación University Hospital Medellín Colombia

**Keywords:** diagnosis, dysphagia, multidisciplinary care, pediatrics, rehabilitation, swallowing disorder

## Abstract

Multidisciplinary clinics bring together multiple specialists to care for patients with complex diseases, such as swallowing disorders. Little information exists regarding dysphagia in pediatric patients and its multidisciplinary management in Colombia. This study aimed to characterize patients under the age of 18 with swallowing disorders treated in a multidisciplinary dysphagia clinic at San Vicente Fundacion Hospital in Medellin, Colombia, between 2014 and 2021. A longitudinal, descriptive, observational study with retrospective data collection was conducted. Sociodemographic and clinical data were obtained at three points. A total of 293 patients were included. The most frequent underlying diseases were neurological (mainly cerebral palsy), followed by genetic disorders, with Down syndrome being the most representative. More than 50% of the patients had been hospitalized at least twice for dysphagia. The diagnosis was primarily clinical, supported by a videofluoroscopic swallowing study. The primary prescribed intervention was speech therapy, followed by an alternate feeding route. This study described pediatric patients with swallowing disorders seen in a multidisciplinary clinic. This overview provides healthcare personnel with an understanding of the complexity of swallowing disorders and their relationships to various medical conditions in children.

## Graphical abstract

This study allowed the characterization of pediatric patients with swallowing disorders seen in a multidisciplinary clinic. This overview provides healthcare personnel with an understanding of the complexity of swallowing disorders and their relationships to various medical conditions in children.

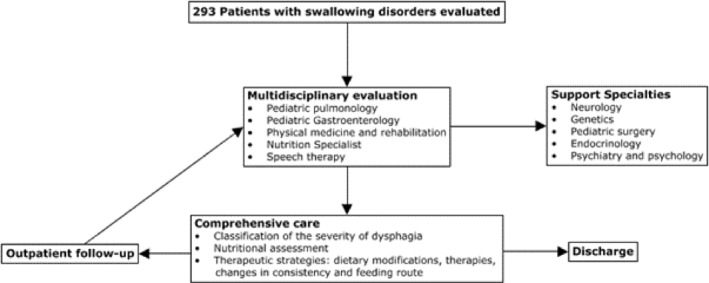

## 1 INTRODUCTION

Feeding is a basic need and a fundamental process of daily life. Swallowing comprises a complex mechanism in which structures from different systems (digestive, respiratory, and neurological) participate in coordination. The term dysphagia includes disorders that affect any of the three phases of swallowing (oral, pharyngeal, and esophageal). It is associated with anatomical, functional, and iatrogenic causes, leading to a broad spectrum of alterations, with swallowing efficiency and safety impairment.^1^

The global prevalence of dysphagia has been increasing in recent years due to various factors.^2^ It affects between 33% and 83% of patients with craniofacial malformations and 33%–80% of children with developmental disorders, reaching figures as high as 90% in patients with childhood cerebral palsy.^3^

In Colombia, the survival of low‐weight and preterm newborns (PTNBs) has increased. This population has a greater need for invasive airway management, which predisposes to a decrease in protective reflexes, atrophy of the laryngeal muscles, and incoordination of the glottic reflex, which, added to immaturity, increases the predisposition to dysphagia,^1^, ^4^, ^5^, ^6^, ^7^ reported in 10.5% of PTNBs and up to 24.5% of PTNBs weighing less than 1500 g.^8^, ^9^ Additionally, swallowing disorders can be found in up to 45% of the healthy population.^1^, ^10^, ^11^, ^12^

Dysphagia has negative impacts on children's health, such as malnutrition (25%–50%), recurrent pneumonia (20%–40%), suffocation crisis (50%), and chronic lung disease secondary to aspiration (12%), as well as disability, poor quality of life for the patients and their families, and a high economic impact on health care.^12^, ^13^ Despite the multiple inherent underlying mechanisms and the need for multidisciplinary care, the diagnosis and treatment of dysphagia have been approached unilaterally, with each discipline suggesting its own approach.^14^, ^15^

This scenario motivated the creation of the dysphagia clinic at Hospital San Vicente Fundacion (HSVF) in 2014, which is a multidisciplinary team covering various medical specialties (gastroenterology, pulmonology, clinical nutrition, speech therapy, and physical medicine and rehabilitation), aimed at offering comprehensive management, with timely and appropriate diagnosis and treatment of swallowing disorders of diverse etiology.

Evidence has shown the benefits of a multidisciplinary approach in highly complex pathologies such as cystic fibrosis and cancer, achieving more timely diagnoses and treatments, greater adherence to medical recommendations, a greater sense of satisfaction, fewer short‐ and long‐term complications, lower costs for the patient and healthcare system, fewer visits to emergency services, and lower expenses due to disability in this population.^16^, ^17^, ^18^, ^19^, ^20^, ^21^

In Latin America, there are no reports of this care modality for pediatric patients with dysphagia. Instances of this modality are scarce in high‐income countries, being more frequent in older adults with neurological diseases and head and neck cancer. In these cases, a significant reduction in the incidence of aspiration pneumonia, improved nutritional status, and greater satisfaction have been demonstrated.^22^, ^23^, ^24^, ^25^

The objective of this study was to describe the experience of a pediatric dysphagia clinic in a tertiary care hospital in Colombia by characterizing the patients treated from 2014 to 2021. This information is the starting point for implementing patient care improvement plans for this group and proposing new research projects.

## 2 MATERIALS AND METHODS

### 2.1 Study design, population, and data collection

A longitudinal, descriptive, observational study was conducted at HSVF, a tertiary care hospital in Medellin, Colombia, using retrospective data collection. The multidisciplinary dysphagia group evaluates around 10 to 15 patients monthly, with simultaneous participation of the nutrition service, physical medicine and rehabilitation, speech therapy, pediatric gastroenterology, and pediatric pulmonology. Patients are referred to this group at the discretion of the treating specialties.

The inclusion criteria were patients under 18 years of age treated at HSVF between 2014 and 2021, with an R13X (dysphagia) ICD10 diagnosis in the electronic medical records, and who were evaluated by the multidisciplinary dysphagia group. Patients in the dysphagia group records were also included. There were no exclusion criteria.

The electronic medical records for all patients were reviewed. Sociodemographic data such as age, sex, and residence; clinical and diagnostic data, such as signs and symptoms, physical examination findings, underlying diseases, and associated comorbidities; and therapeutic indications (for operational definitions of the variables, please see the supplement) were collected. These data were obtained at three points in time: the initial dysphagia clinic office visit, the next available follow‐up, and the last office visit available at the time of the medical record review.

The anthropometric data measured at each office visit were collected. Nursing staff performed these measurements using suitable equipment with INVIMA (Colombian Food and Drug Surveillance Agency) registration. The anthropometric nutritional status classification followed the Colombian Ministry of Health and Social Protection guidelines in Resolution 2465 of 2016. For patients under five years of age, the indicators used were height for age and weight for height, and in patients over five years of age, height for age, body mass index, and body mass index for age. The information was collected in a Microsoft® Excel® version 2016 database, generated from a “Google Forms” format created by the researchers, which included the variables of interest.

### 2.2 Data analysis

A descriptive analysis was carried out in which absolute frequencies and proportions were calculated for categorical variables, and the mean or median for numerical variables with their respective dispersion measures (standard deviation or interquartile range) according to the normality of the variable evaluated by the Shapiro–Wilk test. Statistical analyses were performed using RStudio software, version 2022.07.2.

## 3 RESULTS

We included 293 patients who met the inclusion criteria after an exhaustive review of the electronic medical records, crossing said data with the physical records (See Algorithm 1 and 2 in the Supporting Information S1). The median age at the first visit was 14 months, with 45% of patients younger than 12 months. Patients were most often from urban areas. Regarding perinatal history, 135 (46%) had complications during their gestation (Table 1).

**TABLE 1:** Sociodemographic characteristics.

Variable	*n* = 293
Male, *n* (%)	172 (59)
Age (months), median (IQR)	14 (6–39)
Place of residence, *n* (%)	
Urban	222 (76)
Rural	50 (17)
No data	21 (7)
Residence, *n* (%)	
Medellin	150 (51)
Other municipalities of Antioquia	105 (36)
Outside Antioquia	26 (9)
No data	12 (4)
Gestational age (weeks), *n* (%)	
Greater than or equal to 37	169 (58)
Under 37	100 (34)
No data	24 (8)
Birth weight (grams), *n* (%)	
Greater than 2500	139 (47.5)
Less than 2500	88 (30)
No data	66 (22.5)

Variable	*n* = 135
Perinatal complications, *n* (%)	
Fetal suffering	40 (29.6)
Intrauterine growth restriction	28 (20.7)
STORCH	27 (20)
Hypertensive disorder of pregnancy	27 (20)
Gestational diabetes	13 (9.6)
Premature rupture of membranes	11 (8.1)
Perinatal infection	10 (7.4)
Placental disorders	8 (5.9)
Oligoamnios/Polyhydramnios	7 (5.1)
Kernicterus	1 (0.74)

Abbreviations: IRQ, Interquartile range; STORCH, Maternal infection during pregnancy due to measles, toxoplasma, rubella, cytomegalovirus, herpes virus, human immunodeficiency virus.

### 3.1 Clinical characteristics and diagnostic methods

Regarding associated medical conditions, the most frequent group of underlying diseases was neurological, with infantile cerebral palsy being the primary pathology. The second most frequent was the group of genetic disorders, in which the most frequently reported was Down syndrome (Table 2).

**TABLE 2:** Associated medical conditions.

Variable	*n* = 293
Underlying disease	*n* (%)
Neurological	152 (49.5)
Child brain paralysis	43 (28.3)
Infections	24 (15.8)
Brain/Cranial malformation	23 (15.1)
Hypoxic ischemic encephalopathy	15 (9.9)
Degenerative (myopathies)	13 (8.6)
Perinatal asphyxia	12 (7.9)
Hypotonia	6 (3.9)
Suction‐swallow incoordination	5 (3.3)
Cerebrovascular event	5 (3.3)
Central nervous system trauma	3 (2.0)
Central nervous system tumors	3 (2.0)
Genetics	72 (24.5)
Down's syndrome	31 (43.1)
Inborn error of metabolism	12 (16.7)
DiGeorge	4 (5.6)
Other	46 (63.9)
Gastrointestinal	21 (7.1)
Obstructive malformation	7 (33.3)
Esophageal atresia/Tracheoesophageal fistula	6 (28.6)
Post‐surgical complication	5 (23.8)
Necrotizing enterocolitis	5 (23.8)
Craniofacial	13 (4.4)
Comorbidities	*n* (%)
Neurological	183 (62.2)
Neurodevelopmental delay	108 (59)
Epilepsy	74 (40.3)
Spina bifida	5 (2.7)
Respiratory	181 (61.7)
Broncho obstructive syndrome	68 (31.6)
Pulmonary hypertension	32 (17.7)
Airway malformations	29 (16)
Bronchopulmonary dysplasia	28 (15.5)
Asthma	14 (7.7)
Others	10 (5.5)

For clinical history, 33.5% of the patients had a surgical history, 52% of which had undergone gastrointestinal surgeries, 20.4% cardiovascular interventions, and 15.3% neurological interventions. Of the total number of patients, 89.8% had previously been hospitalized for problems related to swallowing disorders, 53.9% required between 2 and 5 hospitalizations, 12.6% between 6% and 10%, and 4.8% more than 10 hospital admissions, with respiratory diseases being the leading cause of hospitalization in 80% of cases.

Regarding the patients' clinical characteristics on the admission office visit (Table 3), the most affected system was the respiratory system, with abnormal lung sounds in 107 patients (76.4%), decreased respiratory sounds in 44 patients (31. 4%), and signs of respiratory distress in 31 patients (22.1%).

**TABLE 3:** Clinical findings and diagnostic method on admission.

Characteristics	*n* = 293
Feeding consistency, *n* (%)	
Liquid	110 (38)
Thick liquid	50 (17)
Thick	54 (18)
Soft solid	7 (2.5)
All	68 (23)
Fluid restriction	1 (0.5)
No data	3 (1)
Feeding route, *n* (%)	
Oral	155 (53)
Tube	71 (24)
Oral and gastrostomy	27 (9)
Gastrostomy	20 (7)
Oral and tube	18 (6)
Parenteral	2 (1)
Abnormal findings on physical examination, *n* (%)	
Respiratory	140 (47.8)
Gastrointestinal	43 (14.7)
Neurological	187 (63.8)
Diagnosis of dysphagia, *n* (%)	*n* = 273
Clinical	93 (31.6)
Clinical + videofluoroscopic swallowing study	152 (51.4)
Videofluoroscopic swallowing study	28 (10.8)
Videofluoroscopic swallowing study findings, *n* (%)	*n* = 188
Normal	9 (4.8)
Oral phase alteration	119 (63.3)
Pharyngeal phase alteration	155 (82.4)
Esophageal phase alteration	10 (5.3)

With reference to diagnostic aids, videofluoroscopic swallowing studies (VFSS) showed that the pharyngeal phase was the most affected, with mild involvement in 11.7%, moderate in 41.4%, and severe in 29.3%. Joint involvement of the oral and pharyngeal phases occurred in 52%. Anthropometric parameters upon admission were altered in 67.2% of the patients evaluated (Table 4).

**TABLE 4:** Anthropometric characteristics upon admission.

Characteristic	
Height/age classification, *n* (%)	*n* = 276
Adequate	85 (31)
Risk of short stature	66 (24)
Short stature for age	125 (45)
Weight/height classification, *n* (%)	*n* = 247
Appropriate	108 (44)
Risk of acute malnutrition	53 (21)
Moderate acute malnutrition	32 (13)
Severe acute malnutrition	32 (13)
Risk of overweight	4 (2)
Overweight	13 (5)
Obesity	5 (2)
BMI/age classification, *n* (%)	*n* = 35
Appropriate	15 (43)
Thinness	11 (31)
Risk of thinness	4 (11)
Overweight	1 (3)
Obesity	4 (11)

Abbreviation: BMI: body mass index.

### 3.2 Treatment and monitoring

Concerning treatment (Table 5), the primary intervention was speech therapy, followed by prescribing an alternative feeding route in 74% of the patients and changing the consistency of the diet, restricting liquids in 47.7% of the cases. Seven patients (2.4%) were referred for antireflux surgery. Due to adequate progress, the removal of 50% of the gastrostomies and 62.5% of the nasogastric tubes was ordered during follow‐up.

**TABLE 5:** Clinical, imaging characteristics and therapeutic behaviors indicated at admission and during follow‐up.

Characteristics	Admission (*n* = 293) *n* (%)	Fist follow‐up (*n* = 119) *n* (%)	Last follow‐up^c^ (*n* = 54) *n* (%)
Abnormal findings on physical examination			
Respiratory	140 (47.8)	33 (28)	7 (13)
Gastrointestinal	43 (14.7)	10 (8)	3 (5.6)
Neurological	187 (63.8)	60 (51)	24 (44.4)
Videofluoroscopic swallowing study findings	*n* = 188	*n* = 20	*n* = 10
Normal	9 (4.8)	5 (25)	4 (40)
Oral phase alteration	119 (63.3)	5 (25)	3 (30)
Pharyngeal phase alteration	155 (82.4)	14 (70)	5 (50)
Esophageal phase alteration	10 (5.3)	0 (0)	0 (0)
Dietary modification			
Thick diet/liquid consistency restriction	140 (47.7)	62 (21.2)	9 (16.6)
Nasogastric tube			
Insert	102 (34.8)	2 (1.7)	0 (0)
Keep		15/37 (40.5)^a^	3/8 (37.5)^a^
Withdraw		22/37 (59.5)^a^	5/8 (62.5)^a^
Gastrostomy			
Insert	115 (39.3)	7 (5.9)	0 (0)
Keep		34/36 (94.5)^b^	9/18 (50)^b^
Withdraw		2/36 (5.5)^b^	9/18 (50)^b^
Speech therapy			
Yes	221 (75.4)	94 (79)	31 (57.4)
Follow‐up			
Discharge	38 (13)	20 (17)	31 (57.4)
Control	244 (83)	99 (83)	23 (42.6)
Hospitalization	11 (4)	0 (0)	0 (0)

^a^ Calculated on the total number of patients using nasogastric tubes at the time of evaluation.

^b^ Calculated on the total number of patients using gastrostomy at the time of the evaluation.

^c^ Last consultation available at the time of review of the medical history.

On the other hand, most patients evaluated in the first office visit were instructed to continue follow‐up. Evaluation by other specialties was ordered for 57% of the patients, such as neurology (13.3%), genetics (6.5%), ENT (5.2%), occupational therapy (10.6%), speech therapy (14%), respiratory therapy (4.4%) and psychology (2.4%). In the last available office visit, 57.4% of the 54 patients evaluated had not required new hospital admissions since entering the multidisciplinary group.

Regarding orders for additional diagnostic studies in the first office visit, blood chemistries were requested for 7.2% of the patients, imaging for 14%, Videofluoroscopic Swallowing Study (VFSS) for 10%, and chest x‐rays for 2.4%. Other types of studies were requested in 9.5% of cases, including polysomnography (3%), fiberoptic bronchoscopy (1.7%), upper gastrointestinal endoscopy (1.4%), echocardiogram (1.4%), fiberoptic nasopharyngoscopy (0.7%), esophageal manometry (0.7%), and fiberoptic endoscopic evaluation of swallowing (FEES) (0.3%).

Of the total number of patients initially evaluated, the multidisciplinary group discharged 81 patients (27.6%), 128 patients (43.7%) are still being followed, and 84 patients (28.7%) were lost to follow‐up.

## 4 DISCUSSION

To our knowledge, this is the first study to describe the experience of a pediatric dysphagia clinic in Latin America. We found that the patients' primary underlying disease was neurological. Almost 90% had required prior hospitalization for a swallowing disorder. The diagnosis was based mainly on the clinical picture, supported by VFSS, in which the involvement of the pharyngeal phase predominated. The majority of patients had anthropometric abnormalities on admission. The primary intervention ordered was speech therapy, followed by an alternative feeding route.

A median age of 14 months was evident at the first consultation, in accordance with what was found in studies of dysphagia in pediatric patients, such as those carried out by Rommel et al. and Jung et al.^26^, ^27^ Prematurity, as well as low birth weight, were two of the most frequently found antecedents and were pathophysiologically related to dysphagia, as reported by Senekki‐Florent et al.^28^

The most prevalent underlying pathology was neurological, particularly infantile cerebral palsy, followed by the group of genetic disorders, with Down syndrome being the most frequent, data that have also been previously described.^29^, ^30^ This differs from what Dewan et al. reported in adults evaluated at a multidisciplinary dysphagia clinic in California, in whom the most prevalent pathologies were esophageal disorders, followed by head and neck radiation.^31^ However, the high prevalence of respiratory comorbidities in our study is striking, since nearly two‐thirds of the patients evaluated had associated pathologies such as bronchopulmonary dysplasia, asthma, or recurrent bronchial obstruction syndrome.

The basis of the diagnosis of dysphagia is usually the presence of signs and symptoms, which are heterogeneous, affect multiple systems, and vary depending on the patients' underlying disease and comorbidities.^1^, ^32^ In our study, we highlight that most patients had clinical manifestations, with the respiratory system being the most affected, both on the initial physical examination and in the systems review. More than half of our patients had a productive cough with liquids and almost a quarter had recurrent pneumonia. These findings may be related to aspiration events that compromise the integrity of the respiratory system.^33^, ^34^

Other clinical signs found in our patients, and that have been described in the literature as classic for suspecting dysphagia, include accumulation of food in the mouth, previous spillage of food, salivation, rejection of food, gagging and vomiting during feeding, choking, and increased feeding time (more than 30 min).^1^, ^29^, ^32^ Dysphagia should be suspected in children with recurrent respiratory tract infections or unexplained respiratory complications, even if they do not present clinical symptoms or risk factors,^32^, ^34^ as occurred in 15% of our patients. This is consistent with what was evidenced in the study by Lefton‐Greif et al., in which 58% of children with unexplained respiratory problems had silent aspiration of liquids into the airway during swallowing in the absence of known risk factors.^34^

In our study, two‐thirds of the patients had a VFSS on the first evaluation, with the most frequently found abnormality being joint involvement of the oral and pharyngeal phases in 52% of the cases. This agrees with the findings of Ortiz et al.,^29^ in which 78.4% of the patients evaluated had an abnormal VFSS, with combined involvement of the oral and pharyngeal phases in 66.6% of the cases. One of the most critical aspects of VFSS is the visualization of aspirations to the airway, observed in half of the cases in our study, consistent with the study by Romero et al.,^10^ in which 59% of the studies evidenced aspiration in the airway.

The management of children with swallowing disorders may vary depending on the clinical setting and training of medical personnel. The complex nature of this disease warrants a multidisciplinary approach.^30^, ^33^ As described by Dewan et al. and Nandwani et al., the structure of a multidisciplinary dysphagia clinic in California includes evaluation by a laryngologist, gastroenterologist, and speech‐language pathologist. On the same day, they also perform VFSS, FEES, and other tests such as calculating the Eating Assessment Tool (EAT‐10) score.^31^, ^35^ This example shows how the population's objectives, resources, and needs can differ between adult and pediatric clinics.

In our study, the primary intervention was the initiation of speech therapy. To date, there is insufficient evidence for the superiority of any particular type of swallowing therapy, as evidenced in the systematic review by Morgan et al.^36^ Additionally, an alternate route of feeding was ordered during the admission office visit for three out of four patients, higher than what was found by Ortiz et al.,^29^ who noted it in 28.4% of the cases and Romero et al. who reported the need for gastrostomy in 24% of patients.^10^ A reasonable interpretation is that this finding may be related to greater severity of the swallowing disorder and the underlying diseases and comorbidities of the included patients, given that the institution is a regional and national referral center.

Finally, modification of the consistency of the diet was indicated in almost half of the patients, which agrees with the study by Romero et al.,^10^ in which a diet adapted to safer consistencies was prescribed for 56% of the patients. This intervention is based on the fact that thicker liquids reduce the risk of laryngeal penetration and aspiration, as demonstrated in the systematic review by Steele et al.^37^ Regardless of etiology and treatment, a return to a regular diet in children with dysphagia requires a gradual approach to allow systematic neuromuscular training of the pharyngeal phase of swallowing.^33^, ^38^

Little is reported in the literature on the follow‐up of patients with dysphagia, and it is often adapted to the case, etiology, and treatment.^30^ In our study, most patients evaluated on the first office visit were instructed to continue follow‐up, with a median of 3 months between visits, shorter than described in other studies.^29^ More than half of the patients were referred for evaluation by different specialties, which reinforces the complexity of the patients treated.

In the last follow‐up, it was evident that more than half of the patients had not required new hospital admissions, consistent with the findings in other similar studies.^10^, ^29^ Likewise, on the last office visit, the withdrawal of alternative feeding routes was ordered for more than half of the patients, a higher percentage than reported by Ortiz et al. (31.3%).^29^ Approximately a third of the patients did not attend a follow‐up office visit, which drives the search for strategies to improve adherence to the recommendations given by the multidisciplinary group.

### 4.1 Strengths and limitations

One of the outstanding strengths of this study is that it is potentially the first study in Latin America to describe the clinical and demographic characteristics of pediatric patients with dysphagia, as well as their evolution during multidisciplinary management and follow‐up, after having carried out an exhaustive review of the literature.

The limitations of this study are related to possible selection and measurement biases. Potential selection bias was identified when going from 1208 patients with a diagnosis of dysphagia to only 293 patients included in the study because they were evaluated by the multidisciplinary dysphagia group. The sources of information for this study were the electronic medical records and the physical care records of the multidisciplinary dysphagia group, both of which were exhaustively reviewed with double verification by the researchers (SJGB and VAMM). Thus, patients who did not have an R13X (dysphagia) diagnosis in their electronic medical records, but another diagnosis derived from their medical condition (such as their underlying pathology) instead, were captured through the group's manual records. In the same way, patients who had been seen by the multidisciplinary dysphagia group but for some reason did not have a manual form completed were identified by the R13X diagnosis in the electronic medical record, minimizing the possible loss of information and the effect of selection bias. The results cannot be generalized because the study was performed at a single center.

On the other hand, a loss of information was also identified in patients for whom follow‐up was indicated and, for whatever reason, did not return for follow‐up. In Colombia, the provision of healthcare services changes constantly, which can be a factor that influences follow‐up since patients must change the location of care. Thus, follow‐up in the same center is not easy to carry out. Although the leading causes of this loss to follow‐up are unknown, strengthening communication between members of the multidisciplinary group and families, mainly to clarify the importance of periodic assessment, may be a point to work on to increase adherence to management guidelines and adequate follow‐up. This loss to follow‐up could change the population characteristics, leading to selection bias because the patients with more frequent follow‐ups may be sicker than those without adequate follow‐up, so their healthcare provider guaranteed their stay in the clinic.

Given that the anthropometric data were taken from the medical records, there is potential measurement bias. Although the measurements were carried out using suitable equipment with INVIMA registration and periodic calibration, they were taken by the nursing staff on duty, which implies inter‐operator variability.

## 5 CONCLUSION

In conclusion, this study characterized pediatric patients with swallowing disorders treated in a multidisciplinary clinic. Underlying neurological disease and respiratory comorbidities were common. A significant impact on patient morbidity was evident, with a high percentage of hospitalizations related to swallowing disorders before the first office visit. Clinical manifestations were essential for the diagnosis, with predominant neurological and respiratory abnormalities on physical examination, and anthropometric abnormalities in most patients. Video swallowing was a frequently used support tool, with a predominant malfunction of the pharyngeal phase. The primary intervention ordered was speech therapy, followed by an alternative feeding route, which was withdrawn in more than half of the patients seen in the last available follow‐up office visit.

We believe that this overview could provide healthcare personnel with an understanding of the complexity of swallowing disorders and their relationships with various medical conditions in children. This information can be the starting point for implementing plans to improve the care of this group of patients, seeking early diagnosis and timely care to reduce complications and improve their quality of life and that of their families.

## AUTHOR CONTRIBUTIONS

**Silvia J. Galvis‐Blanco**: Conceptualization, Methodology, Data Collection, Writing ‐ Review & Editing. **Víctor A. Martínez‐Moreno**: Conceptualization, Methodology, Data Collection, Writing ‐ Review & Editing. **Olga L. Morales‐Múnera**: Conceptualization, Review & Editing. **Alejandra Wilches‐Luna**: Review & Editing. **Claudia L. Losada‐Gómez**: Review & Editing. **Silvia Palacio‐Petri**: Review & Editing. **Ángela M. Castañeda‐Agudelo**: Review & Editing. **Janeth Rosero‐Vélez**: Review & Editing. **Leidy J. Torres‐Pérez**: Review & Editing. **Laura F. Niño‐Serna**: Conceptualization, Methodology, Statistical Analysis, Review & Editing.

## CONFLICT OF INTEREST STATEMENT

The authors of this study declare that they have no conflicts of interest.

## ETHICS STATEMENT

The Hospital San Vicente Fundacion ethics committee approved the study, as recorded in the 23–2021 min. According to the provisions established in the Colombian Ministry of Health's Resolution 8430 of 1993, this study was classified as risk‐free research. The confidentiality of the data obtained and the identity of the patients was guaranteed. Data analysis was done using software with patient codes which could not be connected to patient names. Only the researchers directly involved in the study had access to the personal data obtained.

## Supporting information

Supporting Information S1

## Data Availability

The data that support the findings of this study are available on request from the corresponding author. The data are not publicly available due to privacy or ethical restrictions.
