# Supplementary material for: Improved outcomes of pediatric patients with swallowing disorders through a multidisciplinary dysphagia clinic in a tertiary care children's hospital in Colombia
Source: Pediatr Discov. 2024 Jul 10;2(4):e99. doi: 10.1002/pdi3.99 (PMC12118282; doi:10.1002/pdi3.99)
Supplement: Supplementary file 1 — Supporting Information S1 [file PDI3-2-e99-s001.docx]

**SUPPLEMENTARY MATERIAL**

**Algorithm 1. Flowchart of the patient selection process for the study**

**
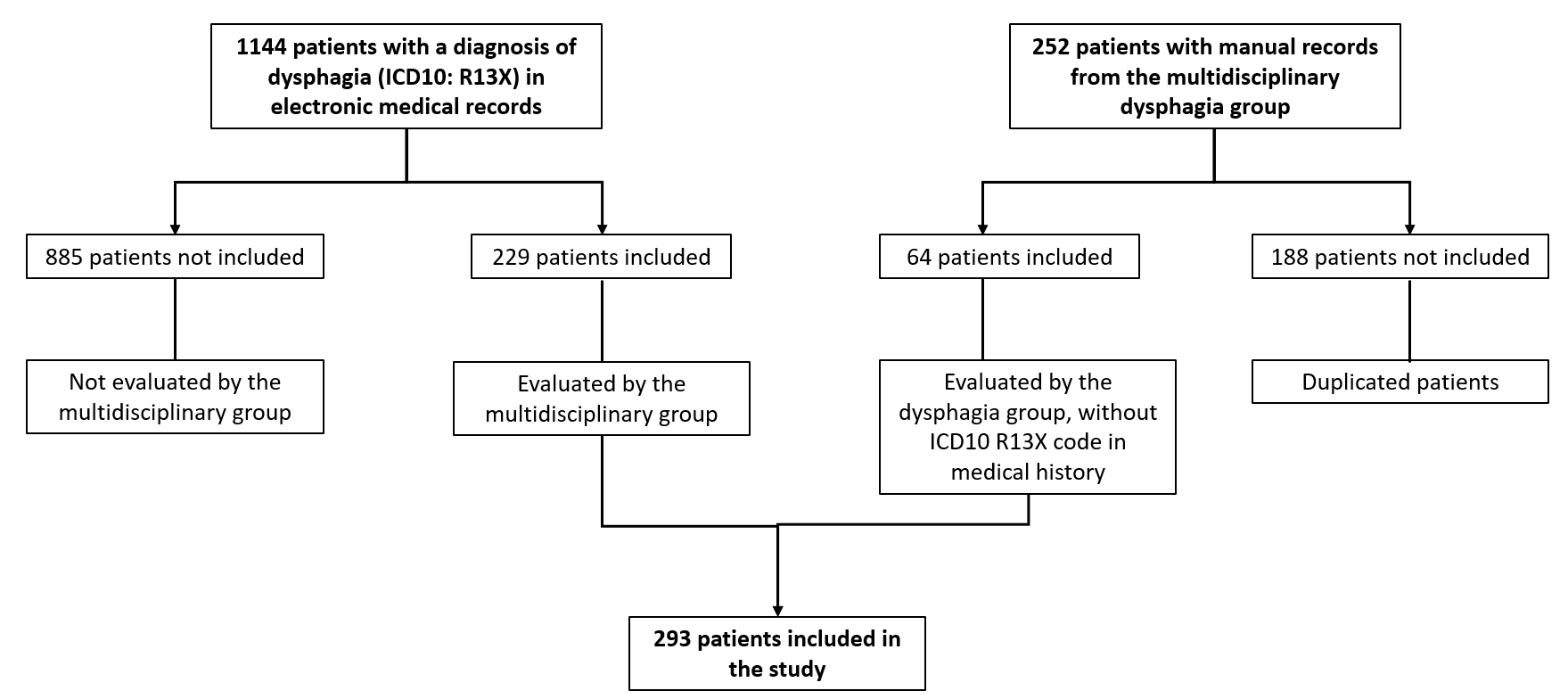
**

**Algorithm 2. Flowchart of patient follow-up during the three consultations.**

**
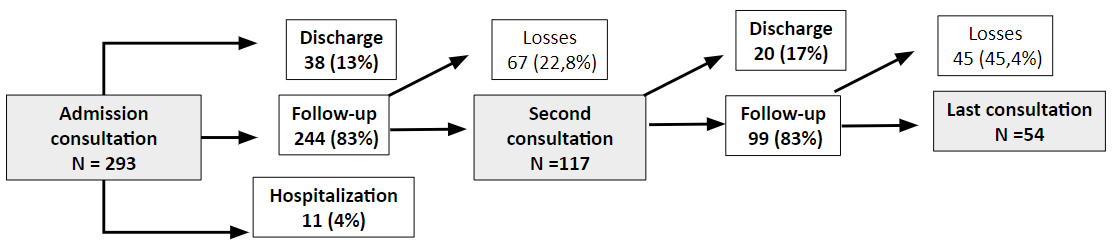
**
